# Public Acceptance and Willingness-to-Pay for a Future Dengue Vaccine: A Community-Based Survey in Bandung, Indonesia

**DOI:** 10.1371/journal.pntd.0002427

**Published:** 2013-09-19

**Authors:** Panji Fortuna Hadisoemarto, Marcia C. Castro

**Affiliations:** 1 Department of Global Health and Population, Harvard School of Public Health, Boston, Massachusetts, United States of America; 2 Department of Public Health, Faculty of Medicine, Padjadjaran University, Bandung, West Java, Indonesia; Institute of Collective Health, Federal University of Bahia, Brazil

## Abstract

**Background:**

All four serotypes of dengue virus are endemic in Indonesia, where the population at risk for infection exceeds 200 million people. Despite continuous control efforts that were initiated more than four decades ago, Indonesia still suffers from multi-annual cycles of dengue outbreak and dengue remains as a major public health problem. Dengue vaccines have been viewed as a promising solution for controlling dengue in Indonesia, but thus far its potential acceptability has not been assessed.

**Methodology/Principal Findings:**

We conducted a household survey in the city of Bandung, Indonesia by administering a questionnaire to examine (i) acceptance of a hypothetical pediatric dengue vaccine; (ii) participant's willingness-to-pay (WTP) for the vaccine, had it not been provided for free; and (iii) whether people think vector control would be unnecessary if the vaccine was available. A proportional odds model and an interval regression model were employed to identify determinants of acceptance and WTP, respectively. We demonstrated that out of 500 heads of household being interviewed, 94.2% would agree to vaccinate their children with the vaccine. Of all participants, 94.6% were willing to pay for the vaccine with a median WTP of US$1.94. In addition, 7.2% stated that vector control would not be necessary had there been a dengue vaccination program.

**Conclusions/Significance:**

Our results suggest that future dengue vaccines can have a very high uptake even when delivered through the private market. This, however, can be influenced by vaccine characteristics and price. In addition, reduction in community vector control efforts may be observed following vaccine introduction but its potential impact in the transmission of dengue and other vector-borne diseases requires further study.

## Author Summary

While methods for vector control such as mosquito breeding source reduction and focal insecticide spraying that have been practiced to reduce dengue transmission in Indonesia have had limited success, dengue vaccines are expected to be an effective control method. However, even if an efficacious vaccine is developed, public acceptance and viable financing mechanisms are crucial for a successful introduction and sustainability of a new vaccination program. In this paper, we report public acceptance and willingness-to-pay for a hypothetical dengue vaccine for children that would be made available in the future. We found a very high proportion of surveyed participants, more than ninety-four percent, were willing to accept and pay for the vaccine. These findings provide a strong support for a dengue vaccination program. On the other hand, we also found a small possibility of reduction in vector control efforts if a dengue vaccination program is put in place. This could have a potential to increase the transmission of other vector-borne diseases and should be taken into account when introducing a dengue vaccination program.

## Introduction

Dengue is endemic in more than 100 countries and places more than 2.5 billion people at risk [1]. Recent modeling of global dengue burden estimated a total of 390 million dengue infections occur annually [2]. This is almost eight times larger than the World Health Organization (WHO) estimate of 50 million dengue infections annually, of which resulted in hospitalization of 1.5 million cases of Dengue Hemorrhagic Fever (DHF) and Dengue Shock Syndrome (DSS) and a case fatality rate of 2.5% [1].

Indonesia is one of the countries where dengue is hyperendemic and all four serotypes are known to circulate in at least 400 of its 497 districts, with more than 200 million people at risk for dengue infection [3]. Recent modeling estimated about 30 million dengue infections occur in Indonesia every year [2].The presence of all four dengue serotypes possibly contributes to the multi-annual cycle of dengue outbreaks with inter-epidemics seasonal transmission [4], with a trend of increasing number of reported DHF cases. In one of the worst dengue outbreaks that occurred in 2010, more than 150,000 cases of DHF were reported to the Ministry of Health of Indonesia, including more than 30,000 hospitalizations [3].

To control dengue virus transmission, the Indonesian dengue program has been focusing its efforts in community-based mosquito breeding place reduction [5]. The program is famous for the slogan “3M” that stands for covering (*Menutup*) and cleaning (*Menguras*) water containers, and burying (*Mengubur*) discarded water containers. However, control of female *Aedes aegypti* mosquito has proven difficult due to its adaptability to the human-made environment, especially in urban settings where dengue is most prevalent [6].

Vaccines have been proposed as a promising solution to dengue control [7]. As of today, more than ten dengue vaccine candidates are in the development pipeline [7], and at least one candidate tetravalent dengue vaccine is projected to be available in the market within the next five years [8]. The magnitude of dengue problems suggests that Indonesia will benefit from a dengue vaccine, as had been suggested by policy makers in the country [9]. Another reason for including a vaccination strategy is the Expanded Program of Immunization (EPI) that has been running from 1977 [10]. To deliver routine vaccinations against seven diseases (polio, measles, diphtheria, tetanus, pertussis, tuberculosis and hepatitis B), the EPI program is supported by a network of 7,800 community health centers, with more than 250,000 community-organized health posts.

On top of the aforementioned epidemiological and programmatic facts, public acceptance should be taken into account in the light of decreasing public trust on vaccination [11]. This is especially true following the false claim made on the link between autism and MMR [12]. In addition, anecdotal evidence also shows concerns over big business involvement, western conspiracy, and the permissibility to use vaccines according to religious teachings, all of which can affect the decision to vaccinate in Indonesia, a predominantly Muslim country (for example, [13]). Nonetheless, few studies on vaccine acceptance in Indonesia showed more than 90% parental acceptance for HPV and anti-typhoid vaccines [14], [15]. Similar studies for a future dengue vaccine are, however, still unavailable.

Financing a dengue vaccination program may be a challenge for a developing country like Indonesia [16]. Thus far, Indonesia self-finances the EPI program with vaccines produced by a government-owned company [10], [17]. Fully vaccinating an infant with EPI vaccines, however, costs less than US$1.00 and the introduction of new vaccination may increase this cost substantially [18]. Indonesian policy makers suggested that the government could finance a dengue vaccination program if the vaccine price is non-prohibitive, with a maximum recommended price of $0.50 per dose [9]. Hence, private source financing may be needed to supplement public financing. For example, a study from the Philippines suggested a mean willingness-to-pay (WTP) for a dengue vaccine of $27 [19]. Viability of delivering such vaccine in the private sector in Indonesia, however, has not been studied.

Arguably, a vaccination program may be followed by a reduction in mosquito control behaviors. Using analogy from other diseases, a study by Newman et al. (2009) found that 10% of high-risk subjects expressed that they would reduce their condom use had they been vaccinated against HIV, which might lead to an increase of other adverse outcomes of unprotected sex [20]. Should this happen following a dengue vaccination program, community-centered mosquito control efforts may be reduced and may lead to an increase in the transmission of other mosquito-borne diseases, such as chikungunya, a virus that shares common vectors with dengue virus [21]. Outbreaks of chikungunya have been documented in many dengue endemic regions in Indonesia [22], and at least one study found that chikungunya is also circulating year round in the city of Bandung [23].

This study contributes to these discussions through a household interview in the city of Bandung, Indonesia. Bandung (total population: 2.3 million) is the fourth most densely populated city in Indonesia with 14,710 people living per square kilometer. Dengue is known endemic, with transmission pattern similar to that observed in Indonesia as a whole [24]. The largest dengue outbreak in Bandung for the past decade occurred in 2009, with a total of 6,678 DHF cases reported to the Ministry of Health (incidence rate: 276 DHF cases/100,000 people). In addition, circulation of all four dengue serotypes in the city has also been documented [25].

In this study, we assessed the extent to which parents would vaccinate their children, and their willingness to pay for a dose of vaccination, in the case that the vaccine was not provided free of charge by the government. We also assessed eventual changes in current dengue control behavior had there been a dengue vaccination program. Potential modifiable determinants were explored to generate recommendation for policy makers in dengue endemic areas.

## Methods

### Ethic Statement

Ethical approval for the study was obtained from the Harvard School of Public Health (Protocol #19173-101) and Padjadjaran University Faculty of Medicine. Survey participants signed an informed-consent form prior to enrollment even though the study met the criteria for exemption.

### Household Survey

The city of Bandung is divided into 30 sub-districts (*kecamatan*), which are further divided into 151 villages (*kelurahan*). Four villages within two sub-districts were selected in consultation with local officials from the Ministry of Health on the basis of representativeness of the target population for a pediatric dengue vaccine in the city of Bandung, accessibility, cooperativeness of local staff and community members, and availability of community health workers. Within a village, households were systematically sampled, starting from a random house and sampling every fifth house until the quota for each village was achieved. The head of household or his spouse was then invited to participate in the study. Between May and July of 2010, we enrolled a total of 500 participants within two sub-districts, Ujung Berung and Antapani. In sub-district Ujung Berung, we enrolled 123 (24.6%) from Pasanggrahan village and 127 (25.4%) participants from Cigending village.

In sub-district Antapani, interviewers were not able to reach the quota of 125 participants in Antapani Kidul village because of unavailability of community health workers. Hence, the quota for the sub-district was therefore fulfilled by sampling more households in the other village within the sub-district, Antapani Tengah. As the result, Antapani Kidul had only 75 (15%) households interviewed whereas the number of household sampled in Antapani Tengah was 175 (35%).

The interview was conducted in Bahasa Indonesia by final year medical students from Padjadjaran University who were trained for the study. To guide and facilitate access to the community, local community health workers were recruited. All 500 households were interviewed over the course of 5 days.

A questionnaire was developed to record participants' demographic information and to measure knowledge, attitude and practice related to dengue, dengue prevention and vaccination in general, acceptance and willingness to pay for a dengue vaccine and their opinion on whether vector control would be necessary had there been a dengue vaccination program. A pilot study involving 30 participants was conducted prior to the survey in order to validate the questionnaire.

### Socioeconomic Level

An asset index was constructed using Principal Component Analysis (PCA), as suggested by Filmer and Prittchett (1999) to categorize participants' socioeconomic level [26], based on fifteen indicator variables, namely access to piped-water, ownership of flushed toilets, radio, landline phone, refrigerators, personal computers, bicycles, motorcycles, cars, internet connection, whether or not they own the housing unit, having a separate room functioning as kitchen and whether the house is built with non-dirt flooring, roof tiles, and brick walls. The first principal component of asset ownership across households explained 25% of the variability. For each household, the asset index was constructed as the sum of standardized asset scores multiplied by their respective factor loadings. Finally, quintiles of the asset index were calculated; households classified in the 1^st^ quintile are the poorest, while those in the 5^th^ quintile are the least poor.

### Primary Outcomes

Primary outcomes of interest in this study were: (i) participants' acceptance of a future, hypothetical, dengue vaccine; (ii) their WTP for the vaccine, had it not been provided for free; and (iii) whether people think vector control would be unnecessary if the vaccine was available. To elicit acceptance of a future dengue vaccine, it was hypothesized that the vaccine would be 100% safe and protective against dengue and provided free by the government as a single dose injection. Acceptance of vaccine was measured by asking participants to respond to the question “would it be likely for you to vaccinate your children?” in a 5-point Likert-like scale ranging from “very unlikely” to “very likely”.

To assess WTP for the hypothetical pediatric dengue vaccine, participants were given a scenario where a fully protective, single dose, dengue vaccine was available. To elicit the maximum amount of money they would be willing to pay for to vaccinate their children, interviewers went through a list of maximum amount of money in an ascending manner starting from less than 10,000 Indonesian Rupiah (IDR); 25,000; 50,000; 75,000; 100,000 and more than IDR100,000 (equivalent to US$1.1, 2.75, 5.5, 8.25, 11.00 and more than 11.00, using the July 2010 exchange rate; US dollar will be used from this point forward). All prices that a participant agreed to pay for was recorded until either the highest price listed or the amount at which participant was no longer willing to pay for was reached. Stated maximum WTP was converted into intervals of bounds of WTP, called from now on as the true WTP, which lies between the highest price a participant would be willing to pay and the next, higher, listed maximum WTP. For example, if a participant agreed to pay a maximum price of $5.5 but not $8.25, the interval that includes true WTP is assumed to be between $5.5 and $8.25. Options to vaccinate their children only when the vaccine was provided free or not to vaccinate their children at all were also provided.

Lastly, to probe for possible behavior change following a dengue vaccination campaign, participants were provided with a scenario where a dengue vaccination campaign using an effective and safe vaccine had been launched. Their responses were recorded in a 5-point scale from “strongly disagree” to “strongly agree” to the statements “the 3M movement is no longer necessary” and “you are not going to do any dengue prevention anymore”.

### Knowledge, Attitude and Practice Related to Dengue

Participants were asked about their knowledge about the symptoms and prevention of dengue, and about dengue virus transmission. To prevent participants from guessing, interviewers did not read aloud choices of answers provided in the questionnaire. Answers that were not listed in the questionnaire were written down and later recoded to match one or more listed options closest to participants' answer.

Responses to knowledge questions were scored between 0 and 1. For questions that had only one possible correct response, a score of 1 was given for that response and 0 otherwise. On the other hand, a score of 0.5 was given for knowledge about dengue symptoms and *Aedes* breeding places if the participant could mention between 1 to 3 correct answers (out of 12 listed for each question) and a score of 1 was given if more than 3 correct responses were provided. For knowledge of dengue prevention, a score of 0.5 was given if the participant could mention one method and a score of 1 was given if more than one method was named. Participants were given a score of 0.3 for correctly mentioning one of the three M's in 3M, a score of 0.7 for 2 correct Ms, and a score of 1 for correctly mentioning all three components of 3M. A score of 0 was assigned when the answer was “don't know” or when the response was incorrect.

Based on this scoring method, we developed a composite dengue knowledge index by including items that maximized the Cronbach's Alpha value as a measure of internal consistency [27]. The final composite index consisted of eight items (Alpha = 0.71) with possible values ranging from 0 to 8. For a more meaningful interpretation in the subsequent analysis, we categorized knowledge scores into tertiles of “good” when the knowledge index scored greater than 6, “sufficient” (index score 4–5) and “poor” (index score<4).

To measure participants' attitude towards dengue prevention, we asked thirteen 5-point Likert-like questions [28]. Response category scales for statements read by interviewers ranged from “strongly disagree” to “strongly agree” with “undecided” as the mid-point. In the analysis, some of the item responses were reverse-coded such that higher score can be assumed to predispose a better practice of dengue prevention efforts. We then constructed a composite attitude index in a similar manner to the construction of knowledge index. A total of five items were used in the final composite index (Alpha = 0.50) with possible scores ranging from 5 to 25. We further categorized attitude index into tertiles of “low support” (score<12), “somewhat supportive” (score 12–14), and “highly supportive” (score>14).

Similarly, five 5-point Likert-like questions were asked to measure participants' acceptance of vaccination programs. A total of three items were included in the final composite index (Alpha = 0.52) with scores ranging from 3 to 15, where a higher score indicates higher support for vaccination. We categorized the acceptance of vaccination scores into tertiles of “low support” when index score was 11 or lower, “somewhat supportive” (score = 12), and “highly supportive” (score>12).

Participants were also asked to report their practice of dengue prevention. Participants were categorized into tertiles of the number of prevention methods being practiced in the month prior to the interview, namely “no effort” if they reported no dengue control activities, while those who reported 1 effort and 2 or more efforts were categorized as having “low effort” and “high effort”, respectively.

### Methodological Approach

In addition to using descriptive statistics of the primary outcomes and covariates of interest, we employed an ordinal regression with a proportional odds assumption to test for the association between dengue vaccine acceptance and several independent variables. The formal model is given by [29]:
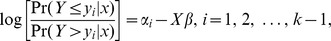
where the left-hand side of the equation is the log odds of being in the *i*-th category for a *k*-category response variable; α*_i_* are the intercept parameters for every category *I*; and *β* is a vector of regression parameters (*β_1_*, *β_2_*, *β_3_*, *…*, *β_j_*) for *X*, the set of *j* explanatory variables in the regression equation.

Because proportional odds ratio model is invariant in magnitude when the coding of outcome variable is reversed, response to outcome variable of interest (dengue vaccine acceptance) was reverse coded such that *e^β^*'s can be interpreted as the odds ratio for having a higher level of acceptance associated with a one unit increase in the dependent variable, holding other variables constant. Variables included as covariates in the model were age, gender, indicators of educational level, and indicators of socioeconomic level (see Table 1); personal experience with dengue, indicators of dengue knowledge level, indicators of attitude towards dengue prevention, indicators of level of support for vaccination and indicators of level of dengue prevention effort. Parameters for the model were estimated using PROC LOGISTIC procedure in SAS 9.30 (SAS Institute, Cary, USA).

**Table 1:** Variables used in the regression analyses.

Variable	Value
**Dependent variables**	
Vaccine acceptance	1 – Unlikely
	2 – Likely
	3 – Very likely
Willingness-to-pay	>IDR0 – <10000
	>10000–25000
	>25000–50000
	>50000–75000
	>75000–100000
	>IDR100000
**Independent variables**	
Dengue experience	0 – No
	1 – Yes
Sex	0 – Female
	1 – Male
Education level	1 – Junior high and lower
	2 – Senior high
	3 – College and higher
Socioeconomic level	1 – Poorest quintile
	2 – 2^nd^
	3 – 3^rd^
	4 – 4^th^
	5 – Richest quintile
Dengue knowledge	1 – Poor
	2 – Sufficient
	3 – Good
Dengue attitude	1 – Weakest
	2 – Middle
	3 – Strongest
Vaccine attitude	1 – Low support
	2 – Supportive
	3 – Highly supportive
Preventive effort	1 – No effort
	2 – Low effort
	3 – High effort
Age (mean)	Centered at mean age, 42.6 years

To estimate the WTP, we excluded those who would not vaccinate their children and those who would vaccinate their children only when the vaccine is provided for free, assuming that lower bound of WTP cannot be less than zero. We then fit an interval regression model to the intervals of the true WTP using PROC LIFEREG procedure in SAS 9.30 [30], [31]. The model is described as:
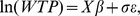
where *ln(WTP)* is assumed to lie in the interval between the log highest price that a participant was willing to pay and the next log highest price stated in the list, *β* is a vector of regression parameters, σ denotes the scale parameter for the distribution, and **ε** is a vector of error terms that are assumed to be normally distributed. The conditional mean and median of predicted WTP can be estimated as 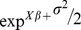 and 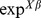, respectively [30]. To examine the associations of different variables with the true WTP, covariates (*X*) included in the WTP model were the same as those included in the previous proportional-odds model.

## Results

### Demographics

More than 80% of participants were female. This may have resulted from the timing of the interview, mostly conducted in weekdays and, to the fact that the household head often delegated participation to his wife. Consequently, most participants reported having no employment because most of them were stay at home mothers. The four villages varied in the socioeconomic distribution of the participants (Table 2). Antapani Kidul and Pasanggrahan had more participants from lower education and socioeconomic levels compared to Antapani Tengah and Cigending. However, between villages comparisons are not of interest to this study, and therefore these differences should not pose a problem on the interpretation of subsequent analyses. Although only a few study participants reported having had prior dengue episode, almost 70% recognized someone who had dengue, reflecting the high dengue incidence in the area.

**Table 2:** Demographic characteristics of participants in each of the villages.

Characteristics	Sub-district Antapani		Sub-district Ujung Berung	
	Antapani Kidul (n = 75)	Antapani Tengah (n = 175)	Cigending (n = 127)	Pasanggrahan (n = 123)
Sex (%)				
Male	9 (12.0)	27 (15.4)	23 (18.1)	18 (14.6)
Female	66 (88.0)	148 (84.6)	104 (81.9)	105 (85.4)
Age	38.0	45.7	43.1	40.7
Education level (%)				
Junior high or lower	58 (77.3)	29 (16.6)	59 (46.5)	83 (67.5)
Senior high	12 (16.0)	96 (54.9)	54 (42.5)	29 (23.6)
College or higher	5 (6.7)	50 (28.6)	14 (11.0)	11 (8.9)
Employment (%)				
Own a business	21 (28.0)	26 (14.9)	20 (15.8)	13 (10.6)
Employee	4 (5.3)	17 (9.7)	13 (10.2)	22 (17.9)
Free lance	8 (10.7)	4 (2.3)	3 (2.4)	12 (9.8)
Not employed	42 (56.0)	128 (73.1)	91 (71.7)	76 (61.8)
Ever had dengue (%)	5 (6.7)	13 (7.4)	7 (5.5)	11 (8.9)
Knows someone who had dengue (%)	29 (38.7)	142 (81.1)	99 (77.9)	79 (64.2)
Socioeconomic level (%)				
Poorest quintile	44 (59.5)	4 (2.3)	16 (12.8)	35 (28.9)
2^nd^	21 (28.4)	14 (8.2)	26 (20.8)	37 (30.6)
3^rd^	3 (4.1)	25 (14.6)	45 (36.0)	26 (21.5)
4^th^	5 (6.8)	52 (30.4)	24 (19.2)	16 (13.2)
Richest quintile	1 (1.4)	76 (44.4)	14 (11.2)	7 (5.8)

### Knowledge of Dengue

More than half of the participants (66.8%) knew that dengue virus is transmitted by mosquitoes. After being informed that dengue virus is transmitted by mosquito bites, 57.3% could mention *Aedes* as the mosquito responsible for the transmission. On the other hand, 68.7% correctly recognized the characteristics of the mosquito responsible for dengue transmission as a black mosquito with white stripes. Finally, having been told about all of the information above, 91.9% correctly identified dengue as predominantly transmitted in daytime.

Fever was the most cited dengue symptom (87.2%) followed by red spots on the skin (ptechiae) (67.6%) and other signs or symptoms. Most participants could mention at least one place of *Aedes* breeding with standing and clean water being the most frequently mentioned by 50.4% and 43.4% of participants, respectively. Components of 3M movement were the most cited dengue prevention methods, although only 8.2% specifically mentioned 3M as one of the methods. Outside of 3M, use of some type of insecticide was also frequently mentioned, as well as fogging or focal spraying and larviciding. Distribution of correct responses for questions measuring dengue knowledge is summarized in Table 3.

**Table 3:** Responses to questions measuring knowledge and dengue prevention practiced in the past month.

	Item #	Question	Correct response (%)*
			(n = 500)
Knowledge	1	Can you mention symptoms of dengue?**	93.4
	2	How is dengue transmitted?	66.9 (n = 498)
	3	What mosquito transmits dengue?	57.3 (n = 499)
	4	How does Aedes mosquito look like?	68.7 (n = 499)
	5	When does Aedes mosquito bite?	91.9 (n = 483)
	6	Where does Aedes breed?	91.6
	7	How can you prevent dengue?**	80.8
	8	What does 3M stand for?**	67.0
Practice	1	Change water in containers	52.6
	2	Cover water containers	22.8
	3	Bury unused containers	20.4
	4	Practice 3 M	11.2
	5	Spray insecticide	10.6
	6	Plug electric insecticide	8.0
	7	Use temephos in water containers	10.0
	8	Apply repellent	7.2
	9	Install window screen	0.2

* Participants were asked about the dengue control and prevention methods that they practiced in the past month.

** Percent with at least one correct response.

### Attitude on Dengue Prevention

Table 2 summarizes the distribution of responses to the five items measuring attitude toward dengue prevention. Most participants considered the city of Bandung as a dengue high-risk area; although most of them did not think that the condition particularly applied to their neighborhood. There was a high agreement on dengue as not being the most important disease in the city, although we did not attempt any further effort to identify what disease participants considered as the most important. Most participants (79.4%) agreed that the government was working hard to prevent dengue. In contrast, there was an overall lack of confidence that dengue prevention could be effectively undertaken by the community or community members.

### Dengue Prevention Practice

Participants were asked to report their practice of dengue prevention in the past week prior to the survey. The most commonly practiced dengue prevention methods were those identified as parts of the 3M movement, although not every component were reported equally often. More than 50% reported that they changed the water inside containers regularly to prevent dengue. In addition, use of insecticides in the forms of sprayed liquid insecticide (10.6%), mosquito coils (5.2%) and insecticide with electric vaporizer (8.0%) were also prevalent. Prevalence of reported methods of dengue prevention practice is described in Table 3.

### Attitude on Vaccination Practice

Five questions were asked to elicit opinion about vaccination (Table 4). In general, there was a very positive attitude about vaccination where the majority of participants agreed that vaccinations were important for disease prevention and that they were safe to be used. The most preferred place to obtain child vaccination was the community integrated health post (27.3%) followed by midwives and community health centers (25.5% and 19.6%, respectively).

**Table 4:** Attitude toward dengue prevention and vaccination (% of total responses).

	Question	Strongly disagree	Disagree	No opinion	Agree	Strongly Agree
Attitude on dengue prevention	Your neighborhood is a dengue high-risk area (n = 499)	4.2	66.9	3.0	24.8	1.0
	The city of Bandung is a dengue high-risk area (n = 498)	1.4	32.5	7.2	55.8	3.0
	The government is doing their best to prevent dengue (n = 500)	0.2	16.6	3.8	73.1	6.4
	You are capable of preventing dengue (n = 497)	2.0	55.7	7.4	34.2	0.6
	Community members are capable of preventing dengue (n = 500)	0.8	59.0	9.8	29.6	0.8
Attitude on vaccination practice	Vaccination is important for disease prevention (n = 500)	0.0	0.0	1.0	73.0	26.0
	Vaccines are safe (n = 500)	0.0	2.8	3.8	85.2	8.2
	You always meet your children's vaccination schedule (n = 499)	0.0	3.8	0.2	83.6	12.4

### Acceptance of Dengue Vaccination

A total of 94.2% of the participants expressed that they were likely or very likely to vaccinate their children. Table 5 shows the proportional odds ordinal regression results with likeliness of vaccinating children as the outcome variable. Due to low number of responses, categories “very unlikely”, “unlikely” and “undecided” for vaccine acceptance were collapsed into one single category. A total of 464 observations were included in the final model.

**Table 5:** Proportional odds ordinal regression results associated with pediatric dengue vaccine acceptance.

Independent variables	n	Unlikely	Undecided	Likely	Very likely	OR (n = 464)	95% CI	*p*-value
Dengue experience	500							
No		5 (3.3)	9 (5.9)	109 (72.2)	28 (18.5)	-	-	
Yes		4 (1.2)	11 (3.2)	223 (63.9)	111 (31.8)	1.9	1.18–2.99	<0.01
Sex	500							
Female		0 (0.0)	3 (3.9)	54 (70.1)	20 (25.9)	-		
Male		9 (2.1)	17 (4.0)	278 (65.7)	119 (28.1)	1.0	0.59–1.85	0.89
Education level	500							
Junior high and lower		4 (1.8)	9 (3.9)	150 (65.5)	66 (28.8)	-	-	-
Senior high		5 (2.6)	10 (5.2)	134 (70.2)	42 (22.0)	0.5	0.27–0.76	<0.01
College and higher		0 (0.0)	1 (1.3)	48 (60.0)	31 (38.8)	1.0	0.53–2.01	0.93
Socioeconomic level	491							
Poorest quintile		2 (2.0)	5 (5.1)	68 (70.1)	22 (22.7)	-	-	-
2^nd^		6 (6.2)	4 (4.1)	69 (68.3)	22 (21.8)	0.8	0.43–1.56	0.55
3^rd^		1 (1.0)	5 (5.0)	59 (62.8)	29 (30.9)	1.4	0.69–2.65	0.38
4^th^		0 (0.0)	2 (2.0)	67 (66.3)	32 (31.7)	1.5	0.73–2.65	0.28
Richest quintile		0 (0.0)	4 (4.3)	64 (65.9)	29 (29.9)	1.4	0.68–3.05	0.34
Dengue knowledge	479							
Poor		2 (1.3)	8 (5.0)	111 (69.4)	39 (24.4)	-	-	-
Sufficient		4 (2.4)	9 (5.4)	110 (65.5)	45 (26.8)	1.0	0.60–1.68	0.98
Good		3 (2.0)	3 (2.0)	99 (65.6)	46 (30.5)	1.3	0.74–2.28	0.35
Dengue attitude	494							
Weakest		4 (3.6)	5 (4.6)	75 (68.2)	26 (23.6)	-	-	-
Middle		4 (1.8)	9 (4.0)	139 (62.3)	71 (31.8)	1.7	1.03–2.95	0.04
Strongest		1 (0.6)	6 (3.7)	114 (70.8)	40 (24.8)	1.4	0.78–2.38	0.28
Vaccine attitude	499							
Low support		2 (4.2)	3 (6.3)	37 (77.1)	6 (12.5)	-	-	-
Supportive		6 (1.9)	12 (3.9)	220 (71.2)	71 (23.0)	2.0	0.94–4.15	0.07
Highly supportive		1 (0.7)	5 (3.5)	74 (52.1)	62 (43.7)	5.0	2.23–11.20	<0.01
Preventive effort	500							
No effort		0 (0.0)	7 (6.5)	73 (67.6)	28 (25.9)	-	-	-
Low effort		5 (3.0)	5 (3.0)	110 (66.7)	45 (27.3)	1.0	0.57–1.72	0.97
High effort		4 (1.8)	8 (3.5)	149 (65.6)	66 (29.1)	0.9	0.52–1.51	0.66
Age (mean)		41.2	39.8	42.3	44.0	1.0	0.99–1.03	0.21

Our model demonstrated that supportive attitude on vaccination practice was the factor most strongly associated with stronger support for dengue vaccination. Compared to participants with low level of support, those within the “somewhat supportive” group of support for vaccination practice were twice more likely to have better support for a dengue vaccine (95% CI: 0.94–4.15, p-value = 0.07) whereas those with a high supportive attitude on vaccination were five times more likely to have a better support for dengue vaccination (95% CI: 2.23–11.20, p-value<0.01).

In addition, personal experience with dengue, whether direct experience from past dengue episode or indirect experience of knowing someone who had dengue, was also strongly correlated with support for dengue vaccination. Participants having personal experience were almost twice more likely to have better acceptance to a dengue vaccine compared to those who did not have personal dengue experience (OR: 1.9, 95% CI: 1.18–2.99, p-value<0.01).

In contrast, individuals who completed a high school education were less likely to support dengue vaccination compared to those with lower education (OR: 0.5, 95% CI: 0.27–0.76, p-value<0.01). This association, however, was not observed among participants with a college degree or higher. Other covariates were not found to be strongly associated with acceptance of dengue vaccine.

**Figure 1:**
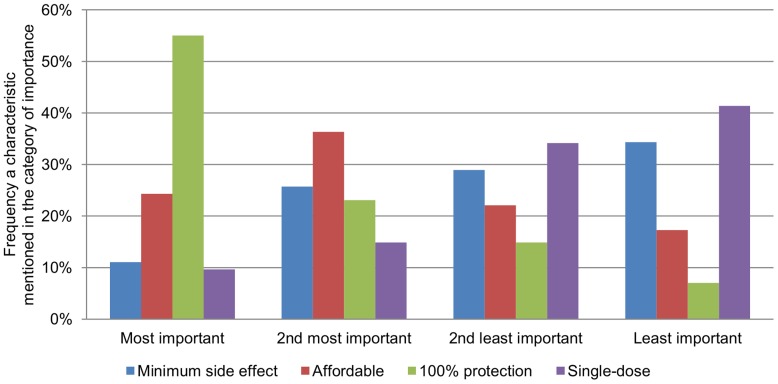
Relative importance of different vaccine characteristics. Participants were asked to rank four characteristics of vaccine according to their relative importance. A majority of participants viewed protection against dengue to be the most important characteristic for future dengue vaccine.

When asked to rank the importance of four vaccine characteristics, more than 50% participants valued full protection against dengue as the most important (Figure 1). This is followed by affordability (36.3%) and safety (26.3%) in the second place whereas the number of doses required for a complete vaccination did not seem to be of great importance relative to the aforementioned factors.

Personal experience with dengue seems to be the only important factor driving non-acceptance in our study. Among those who refused to vaccinate their children, 51.7% had prior experience with dengue, as opposed to 70.9% in the other group (*χ* ^2^ = 4.8, p-value = 0.03). The statistical significance is, however, reduced when other covariates are included in a logistic model (OR: 2.2, 95% CI: 0.978–5.095, p-value = 0.07).

### Willingness to Pay for a Pediatric Dengue Vaccine

**Figure 2:**
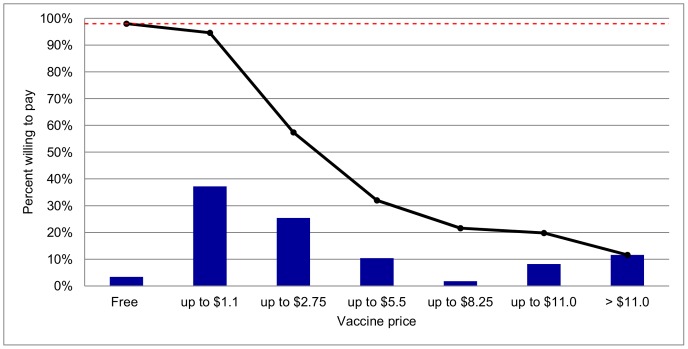
Stated WTP for a hypothetical pediatric dengue vaccine. Bars represent the interval within which the maximum WTP is contained; solid line represents cumulative proportion of participant whose WTP lies below the upper limit of a certain interval. When the vaccine was offered for free, 96.6% of participants (dotted line) were willing to vaccinate their children.

A very small fraction (2.0%) of the participants stated that they would not vaccinate their children even if the vaccine was provided for free, where 3.4% stated that they would vaccinate their children only if the vaccine was provided for free. Among the 94.6% of those willing to pay for the vaccine, a J-shaped distribution of maximum WTP was observed where 37.2% of participants expressed their WTP below $1.1, declining to 1.8% as price went up to a maximum of $8.25 before increasing again to 8.2% and 11.6% for maximum vaccine prices of $11.1 and higher than $11.1, respectively (Figure 2).

We included 438 complete observations in the WTP model and estimated conditional mean and median WTP of $2.64 and $1.94, respectively, for a 42.6 year-old female participant with baseline values of other covariates. The effect of covariates on dengue vaccine WTP in general agreed with our expectation that an increase in educational attainment, socioeconomic status, knowledge, and support for vaccination increases WTP. On the contrary, older participants were willing to pay less compared to their younger counterparts. However, supportive attitude on dengue prevention and higher efforts of dengue prevention were found to be associated with a decrease in WTP (Table 6). Substituting highest covariates values in the model (retaining age at 42.6 years), our model return a maximum predicted conditional mean and median WTP of $13.60 and $9.99, respectively.

**Table 6:** Factors associated with willingness to pay for a pediatric dengue vaccine.

Parameter	Regression Parameter Estimate (n = 438)	95% CI	Mean US$ Estimate	95% CI of US$ Estimate	*p*-value
Intercept	9.8153	9.4128–10.2177	2.64	1.77–3.95	<0.01
Knows someone who had dengue	0.1298	−0.0486–0.3083	+0.37	−0.13–0.95	0.15
Male	0.0375	−0.1917–0.2668	+0.10	−0.46–0.81	0.75
Age (mean centered)	−0.0148	−0.0222–(−0.0075)	−0.04	−0.06–(−0.02)	<0.01
Education level					
Senior high school	0.1047	−0.0973–0.3067	+0.29	−0.25–0.95	0.31
College and higher	0.3470	0.0733–0.6208	+1.10	0.20–2.28	0.01
Socioeconomic level quintile					
2^nd^	0.2471	0.0001–0.4940	+0.74	0.00–1.69	0.05
3^rd^	0.4086	0.1435–0.6736	+1.33	0.41–2.54	<0.01
4^th^	0.5635	0.2800–0.8470	+2.00	0.85–3.52	<0.01
Highest	0.7146	0.4183–1.0109	+2.76	1.37–4.62	<0.01
Dengue knowledge category					
Middle	−0.0071	−0.2068–0.1926	−0.02	−0.49–0.56	0.94
Highest	0.2272	0.0044–0.4499	+0.67	0.01–1.50	0.05
Support on dengue prevention					
Supportive	−0.0818	−0.2897–0.1262	−0.21	−0.67–0.36	0.44
Highly supportive	−0.0591	−0.2775–0.1592	−0.15	−0.64–0.46	0.60
Support on vaccination					
Supportive	0.2205	−0.0570–0.4981	+0.65	−0.15–1.71	0.12
Highly supportive	0.1819	−0.1191–0.4829	+0.53	−0.30–1.64	0.24
Preventive effort					
Low effort	−0.0062	−0.2257–0.2133	−0.02	−0.53–0.63	0.96
Highs effort	−0.0381	−0.2478–0.1716	−0.10	−0.58–0.49	0.72
Scale	0.7863	0.7234–0.8548			

### Potential Behavior Change

Participants were asked if they would give up current dengue prevention efforts or felt that 3M movement would no longer be necessary, should there be a mass dengue vaccination campaign. Most participants disagreed that dengue prevention efforts were no longer necessary. There were, however, 7.0% who thought that 3M movement would no longer be necessary, and 6.8% who expressed that they would not practice dengue prevention anymore. Of these, about 30% reported that they did not practice any dengue prevention in the past month.

## Discussion

Our study sought to explore public acceptance of a hypothetical dengue vaccine, to determine whether participants would be willing to pay for the vaccine and to explore the possibility of behavioral changes following a dengue vaccination program. Our results demonstrated that the hypothetical pediatric dengue vaccine would be accepted by 94.2% of the survey participants. Furthermore, 94.6% expressed their willingness to pay for the vaccine with a median of stated WTP of US$1.94. We also found that 7% of the participants agreed that other dengue prevention methods are no longer necessary once dengue vaccine is available, among which, 30% were not practicing any dengue prevention in the week prior to the survey.

### Acceptance of Future Dengue Vaccine

In this study, we identified the most important determinant of public acceptance of a future dengue vaccine to be parental acceptance of vaccination practice. The supportive attitude on vaccination practice is reflected in national coverage of EPI vaccination of 93.4% among infants and 92.5% among school children, despite lower coverage in some of the eastern parts of Indonesia where health services are less adequate [3]. Likewise, EPI vaccines coverage in Bandung is generally above 95%, with an exception of at birth dose of Hepatitis B vaccine (80.2% coverage) [24]. Arguably, parents living in Bandung are used to the idea of child vaccination due to the extensive vaccination campaigns performed by the government and by the routine vaccination that their children received.

Participants who had personal experience with dengue were also more likely to accept future dengue vaccination (OR: 1.9, 95% CI: 1.18–2.99, p-value = 0.01). This makes sense because these parents were more able to weigh the possible benefits of vaccinating against dengue, given the perceived risk of having their children getting the disease. Similar association was also presented in at least one previous study showing that parents with previous abnormal cervical smear findings were more willing to vaccinate their daughters with HPV vaccine [32]. The same study also found that in the presence of negative personal experience, increasing knowledge about HPV did not have a discernible effect in increasing parental acceptance for HPV vaccination, a result that also comes up in our study.

Alternatively, this high acceptance can be attributed to the perceived barrier to performing dengue prevention. As we have shown, even though more than 90% of participants thought that prevention of dengue was important, only one-third thought that the efforts could be done by individuals or community members. Apparently, Indonesian policy makers also have similar concern that community-based dengue prevention will not work due to competing priorities among the community members themselves [9].

It is very likely that the perceived need for a dengue vaccine will be high. This is arguably conditioned by the high incidence of dengue in the study area and, a high community and media attention to dengue (for example [33]). Thus, dengue has been a constant public concern and a future vaccine is likely to be on a high demand among the population. As was shown in this study, the protective effect of the vaccine was highly valued by the participants.

### WTP for a Dengue Vaccine

For an average individual in our sample, the mean and the median reported WTP for a dengue vaccine was $2.64 and $1.94, respectively. Median WTP is considered more robust to skewness in WTP distribution and hence will be used as the reference measure. Although the stated public WTP seems to be low (average household monthly expenditure in Bandung approximately US$ 200), it agrees with Indonesian policy maker's WTP for such vaccine, which ranged from US$0.5 per dose to US$2.0–3.0 per series [9]. In addition, the result is not surprising considering that all EPI vaccines can be obtained free from public hospitals, community health centers and integrated health posts. Production price of a dengue vaccine has been estimated to be as low as $0.2 per dose [34]. Nonetheless, past experience have shown new vaccines introduced at prices unaffordable to developing countries and that creating sufficient demand to bring the production cost down to an affordable level required substantial efforts and time. In the case of Hepatitis B vaccine, it took 20 years to bring the price down from $30 per dose to approximately $1 per dose, after which its adoption and coverage in developing countries increased significantly [35].

On the other hand, we found a wide range of prices, with a J-shaped distribution, at which the participant would agree to pay for the hypothetical vaccine. With almost 20% of participants expressed their WTP for a pediatric dengue vaccine of $11.0 or more, there could be a market for the vaccine at a higher price. It is, however, impossible to tell how much coverage can be attained through private sector until the vaccine is actually available in the market. Yet, achieving a high coverage in the private sector seems unlikely due to the fact that 45% of Indonesia's population is not covered by any health insurance. Social insurance schemes that cover most of the insured do not usually cover vaccination services either. Because vaccination services in the private market most likely will be obtained through out-of-pocket payment, it is very likely that provision of partially or fully subsidized vaccines will be necessary to achieve large scale coverage.

Our study suggests that wealthier people are more likely to spend more money for the vaccine. Hence, any pricing policy must take into account the possibility of increasing the gap of dengue disease burden between the affluent and the less affluent. In this regard, price tiering and cross-subsidization of vaccine seems to be one financing option. Another option is to advocate the inclusion of vaccination services, or at least dengue vaccination, in the benefit package of the upcoming universal insurance coverage scheme that will be rolled out in the year 2014.

### Potential Behavior Change

Behavior change is often cited as a concern in vaccine studies [20], [36]. In the context of the city of Bandung, an urban area that is also known to be endemic of Chikungunya virus, vector control is necessary even when herd immunity against dengue is achieved. Our study found that about 7% of participants thought that the 3M movement, Indonesia's government current mainstay of dengue prevention, is no longer necessary when a vaccination program is in place. This potential reduction may not have a significant effect on the transmission of other mosquito-borne infectious agents and hence we will interpret this number with caution.

It is known that different types of water containers vary in their capacity to produce adult *Aedes* mosquito [37], [38]. However, container productivity characteristics can differ by regions and hence the impact of source reduction may depend on the identification of these containers. For example, discarded items were found to be the most productive sources of *Aedes* in the city of Gioania, Brazil, whereas in Yogyakarta, Indonesia, the most productive containers were *bak mandi*, large containers commonly used to store water for bathing [39], [40]. But even when source control is applied using this information, unintended dispersion of oviposition place to previously unrecognized containers may occur [41].

Hence, reduction in vector control efforts may or may not produce actual increase in vector population, especially when the reduction is small. Nonetheless, the impact of current vector control strategies on vector population and disease dynamics in Indonesia is understudied and our findings may warrant further attention to the potential effect of vector control reduction.

### Study Limitations

Arguably, it remains unclear whether our findings will translate into actual behavior. Critics argue that stated preference model may suffer from inaccuracy and bias [42]. One important limitation in this study is the portrayal of the hypothetical vaccine as fully efficacious and safe, which would have affected how the participants responded to questions related to the vaccine. This vaccine portrayal might be elusive as was shown from recent evidence from a phase IIb randomize trial in Thailand, in which the tetravalent pediatric dengue vaccine provided an overall efficacy of 30% [43]. Therefore, actual acceptance and WTP for the vaccine could be adjusted by the actual vaccine efficacy. However, our vaccine representation could be regarded as a way to elicit the ceiling for acceptance and WTP such that the actual acceptance and WTP would not exceed what we found from this study. Lastly, our study was conducted only in one city, Bandung, which will not represent the diversity of Indonesia as a whole. Nonetheless, dengue has been recognized as a problem most prominent in urban areas and therefore we believe that Bandung can represent most, if not all, cities in Indonesia where dengue is prominent. The study generated a wealth of information regarding community members' acceptance of a pediatric dengue vaccine in Bandung, and our results can be used as the basis for further research. We specifically propose to validate these findings in a future community pilot, once a dengue vaccine is available. Results from such studies can be used to further model the impact of a dengue vaccination program and will be most useful in assisting policy making.
